# Uncovering the prognostic gene signatures for the improvement of risk stratification in cancers by using deep learning algorithm coupled with wavelet transform

**DOI:** 10.1186/s12859-020-03544-z

**Published:** 2020-05-19

**Authors:** Yiru Zhao, Yifan Zhou, Yuan Liu, Yinyi Hao, Menglong Li, Xuemei Pu, Chuan Li, Zhining Wen

**Affiliations:** 1 College of Computer Science, Sichuan University, Chengdu, 610064 Sichuan China; 2 College of Chemistry, Sichuan University, Chengdu, 610064 Sichuan China; 3 Medical Big Data Center, Sichuan University, Chengdu, 610064 Sichuan China

**Keywords:** Convolutional neural network, Stationary wavelet transform, Cancer prognosis prediction, RNA-sequencing, Cox regression

## Abstract

**Background:**

The aim of gene expression-based clinical modelling in tumorigenesis is not only to accurately predict the clinical endpoints, but also to reveal the genome characteristics for downstream analysis for the purpose of understanding the mechanisms of cancers. Most of the conventional machine learning methods involved a gene filtering step, in which tens of thousands of genes were firstly filtered based on the gene expression levels by a statistical method with an arbitrary cutoff. Although gene filtering procedure helps to reduce the feature dimension and avoid overfitting, there is a risk that some pathogenic genes important to the disease will be ignored.

**Results:**

In this study, we proposed a novel deep learning approach by combining a convolutional neural network with stationary wavelet transform (SWT-CNN) for stratifying cancer patients and predicting their clinical outcomes without gene filtering based on tumor genomic profiles. The proposed SWT-CNN overperformed the state-of-art algorithms, including support vector machine (SVM) and logistic regression (LR), and produced comparable prediction performance to random forest (RF). Furthermore, for all the cancer types, we firstly proposed a method to weight the genes with the scores, which took advantage of the representative features in the hidden layer of convolutional neural network, and then selected the prognostic genes for the Cox proportional-hazards regression. The results showed that risk stratifications can be effectively improved by using the identified prognostic genes as feature, indicating that the representative features generated by SWT-CNN can well correlate the genes with prognostic risk in cancers and be helpful for selecting the prognostic gene signatures.

**Conclusions:**

Our results indicated that gene expression-based SWT-CNN model can be an excellent tool for stratifying the prognostic risk for cancer patients. In addition, the representative features of SWT-CNN were validated to be useful for evaluating the importance of the genes in the risk stratification and can be further used to identify the prognostic gene signatures.

## Background

For the past decade, the gene expression-based models had been widely used in the cancer researches for predicting the clinical outcomes and made considerable progress [1, 2]. A number of machine learning algorithms had been proposed to construct predictive models and validated in various cancer types [3–7], for the purpose of identifying the genome characteristics, e.g. cancer-related differentially expressed genes or structural variations, as well as predicting the clinical outcomes, such as the risk stratification for the patients in cancers. Although the performance of the predictive models largely depends on the number of samples collected for model training and is restricted by the endpoint predictability to a certain extent [8], the feature selection is also a vital step the gene expression-based modeling in the clinical outcomes prediction. In most cases, genes are firstly filtered by comparing the expression levels between two phenotypic conditions in clinics with a statistical method and using an arbitrary cutoff, e.g. *p* value < 0.05, and then only the rest of genes are applied to the model construction as features. Based on the filtered gene list, a series of variable selection methods, such as stepwise regression [9], simulated annealing [10] and variable combination population analysis (VCPA) [11], are also developed to identify the useful features for model construction [12, 13].

However, considering the fact that the statistics-based method with a ‘hard’ cutoff doesn’t necessarily evaluate the contribution of a gene to the clinical prediction, it is not a reasonable way to filter out genes before model construction, which may result in the omission of a part of genes that are still important to the disease. Therefore, we suggested a deep learning-based strategy as an alternative, which combined the convolutional neural network [14–16] with stationary wavelet transform [17] (SWT-CNN), to predict the survival in different cancer patients by using as many genes as possible to reduce the loss of feature information. In recent years, the emerging deep learning technique [18] has achieved rapid development in image processing field [19] as well as in the related areas, such as voice recognition [20], nature language processing [21] and chemical pattern recognition [22], in virtue of its representation learning strategy [23], which is announced to be superior to the conventional predictive learning because of its powerful ability to generate more complex representations of the target objectives by combining the simple features [24]. As a result, the representation learning algorithms are expected to perform better in variable selection procedure than other conventional methods. Several studies had also utilized the deep learning methods to predict the cancer prognosis with the genomics [25–30] data as well as reported the evaluation of the predictive performance of deep learning methods when compared with conventional machine learning models [31]. These studies only focused on the performance of deep learning algorithms on predicting the clinical endpoints and paid little attention on discussing the contribution of the genes in the prediction procedure, which isn’t conductive to improving predictive results and seeking the key diagnostic gene signatures for better understanding the disease mechanism.

In this study, we proposed SWT-CNN to stratify the prognostic risk for cancer patients by using as many genes as possible and validated it with the gene expression data of multiple cancer types downloaded from The Cancer Genome Atlas (TCGA) database [32]. Based on the evaluation of 15 tumor genomics datasets in TCGA, SWT-CNN provided superior performance compared to support vector machine (SVM) and logistic regression (LR), and yielded a comparable performance to random forest (RF). In addition, we also attempted to extract the representative features from the hidden layers of the CNN for evaluating the importance of the genes in risk stratification and prediction. In fact, when generating representative features, CNN tends to give heavy weights to those features with large values while ignoring some small ones. It is not a problem for image recognition and classification, but it is not suitable for genomics data modeling, because genes with relatively low expression levels may still be closely related to the clinical outcomes. Therefore, we introduced the wavelet transform algorithm, which is successfully used for the gene expression data analysis in previous studies [33–41], to enhance the significance of genes with relatively low expression levels in the gene list, so that CNN can give appropriate weight when abstracting and extracting features. For all the cancer types, we first evaluated the relationship between each gene and clinical outcome by scoring the gene based on the representative features in CNN, then selected those closely related to clinical outcome for the subsequent Cox proportional-hazards regression and prediction. Our results show that compared with SWT-CNN results, the risk prediction is further improved. The median overall survival time of high-risk patients stratified by Cox regression was lower than that of the patients classified by SWT-CNN. It demonstrates that representative features are useful for identifying the diagnostic genes and improving stratification of the cancer patients.

## Results

### Study design

**Fig. 1:**
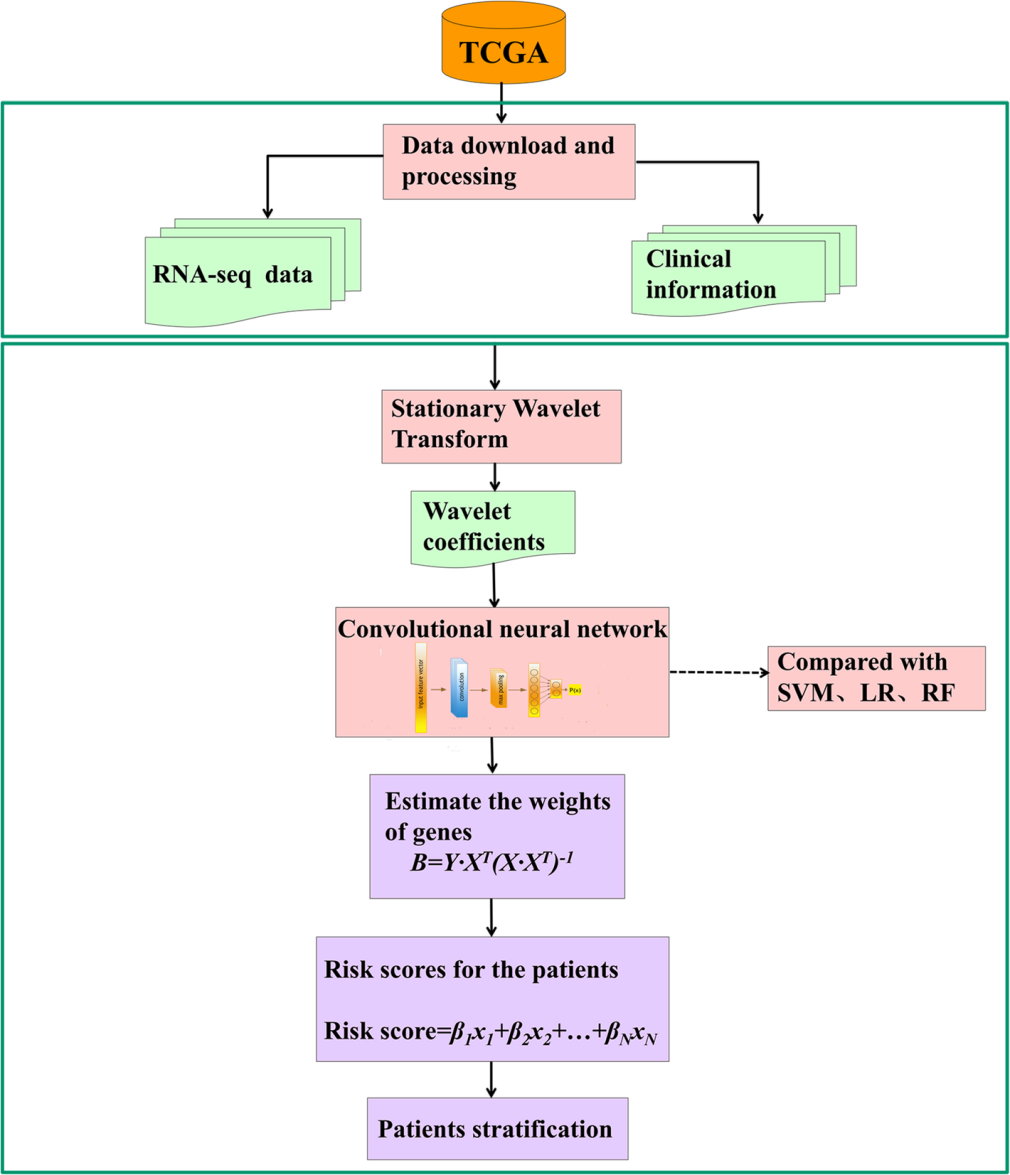
The workflow of our study

In this study, the RNA-sequencing data and the clinical information of all cancer types were collected from The Cancer Genome Atlas (TCGA) [32] database and the patients were categorized into low- and high-risk groups according to their tumor stages and overall survival times respectively. The gene expression profile of each patient was firstly decomposed by SWT, and then the wavelet coefficients were inputted into CNN for predictive model construction. The comparative analysis of the model performance was conducted among SWT-CNN, SVM, RF and LR. More importantly, in order to explore the effectiveness of representative features in identifying diagnostic genes, we proposed a scoring function to estimate the weights of genes based on the representative features extracted from the hidden layer of CNN and selected the gene signatures for stratifying the patients in all the cancer types. The overview of our study was depicted in Fig. 1.

### Selection of wavelet functions

**Fig. 2:**
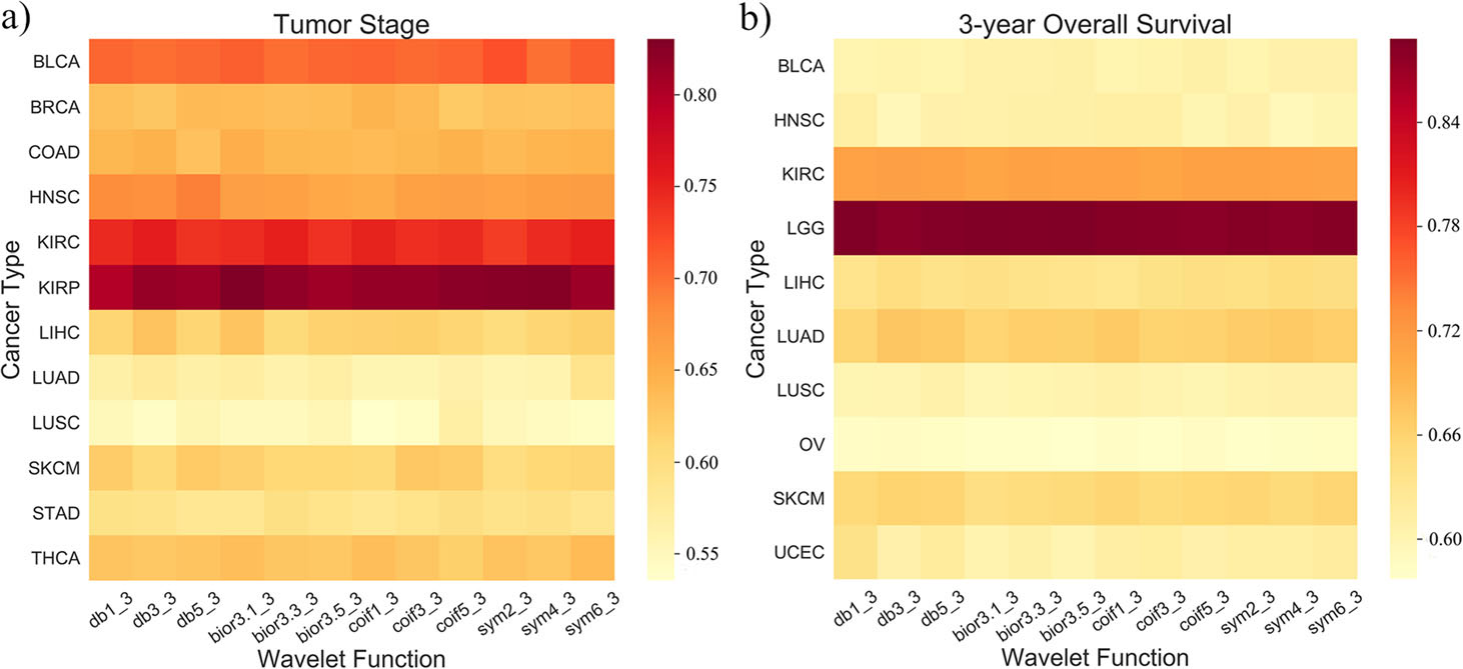
The AUCs achieved by using different wavelet functions for the prediction of tumor stages and 3-year overall survival. **a** The AUCs of predicting the tumor stages across different cancer types in validation set. **b** The AUCs of predicting the 3-year overall survivals across different cancer types in validation set

Considering the fact that different wavelet functions are suitable for different signals and different wavelet coefficients will be generated, we applied 12 commonly used wavelet functions (4 wavelet families × 3 wavelets functions per family) in decomposing the gene expression profiles of the patients and investigated the predictive performance of SWT-CNN. The AUCs of predicting risk stratification on the basis of tumor stages and 3-year overall survivals across different cancer types in validation set were shown in Fig. 2a and b, respectively. It can be seen from Fig. 2 that the difference of AUCs for the prediction of tumor stages and overall survivals mainly existed in different cancer types. For the prediction of tumor stages and 3-year overall survivals, the best AUCs were achieved for KIRP (AUC = 0.83) and LGG (AUC = 0.89), respectively.

In addition, constructing models with the wavelet coefficients decomposed by different wavelet functions also had a certain impact on the prediction results. For each cancer type, we choose the most appropriate wavelet function to predict the tumor stages and overall survivals according to the AUCs. The optimal wavelet function as well as the corresponding best AUC in the prediction of the risk differentiated by tumor stages and 3-year overall survivals were listed in Tables 1 and 2, respectively. When predicting the tumor stages, the performance of CNN models with the wavelet coefficients decomposed by different wavelet functions in the wavelet families is different for 12 cancer types (Table 1). Interestingly, for the prediction of the overall survival after 3 years, more than half of cancer types achieved the best AUC when using *Daubechies* wavelet family to generate the wavelet coefficients (Table 2). Compared with the functions in other wavelet families, the wavelet functions in *Daubechies* wavelet family were simple with minimum support width, indicating that after the decomposition by the *Daubechies* wavelet functions, the fluctuation of the wavelet coefficients is small. Although the magnitude of wavelet coefficients of the original gene expression profiles became weaker after the decomposition by *Daubechies* wavelets, the prediction results of CNN model became better, which meant that *Daubechies* wavelet decomposition can better highlight the expression signals than the functions in other wavelet families when predicting the overall survival. On the contrary, the performance of the functions in the *symlets* wavelet family is relatively poor in predicting tumor stages and overall survival. Finally, as listed in Tables 1 and 2, we used the optimal wavelet function for each cancer type in the subsequent analysis.

**Table 1:** The detailed information of the data sets for tumor stage prediction

Cancer Type	#of all samples	#of samples		Proportion of 1/0 samples	Wavelet Function	AUC
		positive	negative			
BLCA	403	271	132	1:0.49	*sym2*	0.72
BRCA	1055	267	788	1:2.95	*coif1*	0.64
COAD	442	190	252	1:1.33	*bior3.1*	0.65
HNSC	430	336	94	1:0.28	*db5*	0.69
KIRC	524	204	320	1:1.57	*db3*	0.75
KIRP	259	66	193	1:2.92	*bior3.1*	0.83
LIHC	347	88	259	1:2.94	*db3*	0.63
LUAD	505	110	395	1:3.59	*sym6*	0.59
LUSC	492	91	401	1:4.41	*coif5*	0.57
SKCM	424	195	229	1:1.17	*coif3*	0.62
STAD	350	186	164	1:0.88	*coif5*	0.60
THCA	503	167	336	1:2.01	*sym6*	0.64

**Table 2:** The detailed information of the data sets for 3-year overall survival prediction

Cancer Type	#of all samples	#of samples		Proportion of 1/0 samples	Wavelet Function	AUC
		positive	negative			
BLCA	248	161	87	1:0.54	*bior3.5*	0.61
HNSC	311	178	133	1:0.75	*coif1*	0.61
KIRC	400	109	291	1:2.67	*db3*	0.71
LGG	240	79	161	1:2.04	*bior3.1*	0.89
LIHC	196	104	92	1:0.88	*sym4*	0.65
LUAD	267	134	133	1:0.99	*db3*	0.67
LUSC	302	154	148	1:0.96	*db5*	0.61
OV	274	112	162	1:1.45	*db3*	0.59
SKCM	340	112	228	1:2.04	*db3*	0.66
UCEC	279	70	209	1:2.99	*db1*	0.64

### Performance of SWT-CNN on clinical prediction

**Fig. 3:**
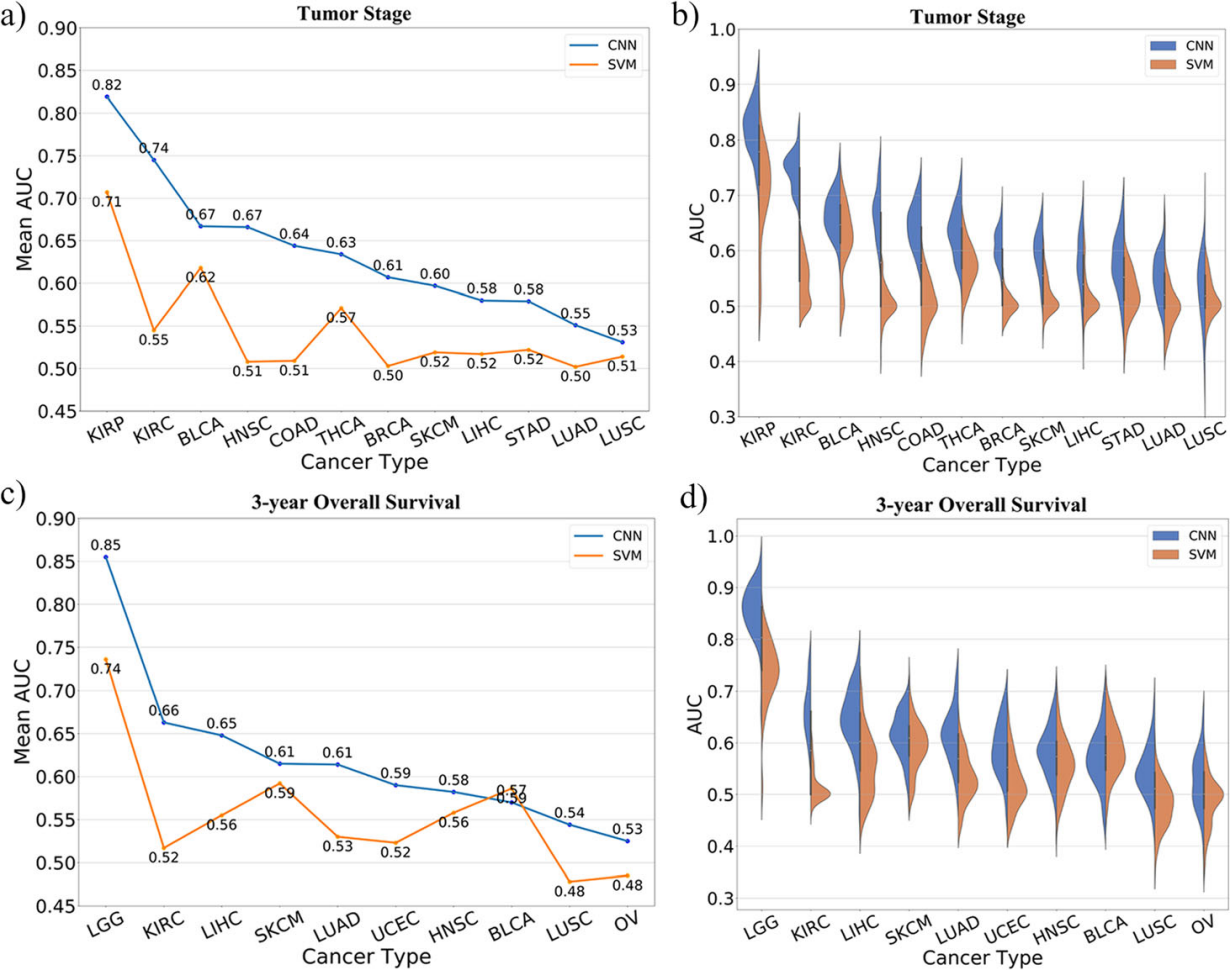
The mean AUCs as well as the distribution of AUCs achieved by SWT-CNN and SVM with 100 sampling times for the prediction of tumor stages and 3-year overall survival. **a** Mean AUCs achieved by SWT-CNN and SVM for predicting the tumor stages. **b** The distribution of AUCs achieved by SWT-CNN and SVM for predicting the tumor stages. **c** Mean AUCs achieved by SWT-CNN and SVM for predicting the 3-year overall survivals. **d** The distribution of AUCs achieved by SWT-CNN and SVM for predicting the 3-year overall survivals

After SWT decomposition, the wavelet coefficients of the gene expression profiles were inputted into a one-layer CNN model, which was announced to be sufficient for gene expression data modeling [31]. The area under the receiver operating characteristic curve (AUC) was used as performance metric for evaluating the predictive models. At the same time, as a comparison, we used SVM to predict the tumor stages and overall survivals. The AUCs achieved by SWT-CNN and SVM for predicting tumor stages of 12 cancer types and 3-year overall survival of 10 cancer types were shown in Fig. 3.

In general, the model performance largely depended on the predictability of the endpoints, which was consistent with previous study [8], even if the RNA-sequencing data was used. The tumor stages of KIRP and the 3-year overall survival of LGG were easier to predict and both mean AUCs achieved by SWT-CNN and SVM were higher than 0.7. By contrast, the tumor stages of LUSC and the 3-year overall survival of OV were the most difficult to predict. Both mean AUCs achieved by SWT-CNN and SVM were near 0.5 (Fig. 3a and c). Compared with the results of SVM, the AUCs achieved by SWT-CNN were higher, except for the prediction of 3-year overall survival of BLCA.

In terms of details, for the easily predicted cancer types, the performance of SWT-CNN was better than that of SVM. It can be seen from Fig. 3a and c, the mean AUCs of predicting the tumor stages of KIRP and the 3-year overall survival of LGG achieved by SWT-CNN (mean AUCs = 0.82 and 0.85, resp.) were 0.1 higher than those achieved by SVM (mean AUCs = 0.71 and 0.74, resp.). For some cancer types that were difficult to predict by SVM, SWT-CNN can still perform better. When predicting the tumor stages of KIRC, the mean AUC achieved by SWT-CNN (mean AUC = 0.74) was 0.19 higher than that achieved by SVM (mean AUC = 0.55). Almost all the AUCs from 100 random sampling achieved by SWT-CNN were higher than those achieved by SVM (Fig. 3b). Similar results can be found in the prediction of the tumor stages of COAD (mean AUCs for SWT-CNN and SVM = 0.64 and 0.51, resp.). As to the prediction of 3-year survival of KIRC, although the prediction results of SWT-CNN (mean AUC = 0.66) were 0.14 higher than that of SVM (mean AUC = 0.52), the AUCs of 100 random sampling achieved by SWT-CNN were scattered (Fig. 3d), indicating that the prediction of this cancer by SWT-CNN might be not stable enough.

**Fig. 4:**
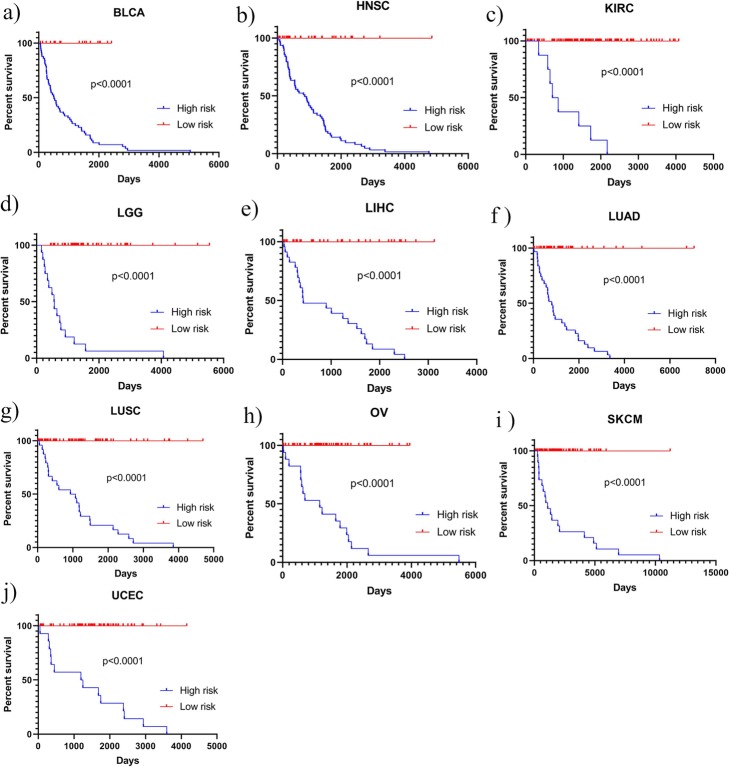
The results of Kaplan-Meier survival analysis of all cancer types

Some cancer types were difficult to predict by both models. When predicting the tumor stages of LUAD and LUSC, the mean AUCs achieved by SWT-CNN were only 0.55 and 0.53, respectively. The prediction results of SVM were similar to random results (mean AUCs = 0.50 and 0.51 for LUAD and LUSC, resp.). Similar results can be found in the prediction of 3-year overall survival of OV (mean AUCs = 0.53 and 0.48 for SWT-CNN and SVM, resp.) and LUSC (mean AUCs = 0.54 and 0.48 for SWT-CNN and SVM, resp.). For such cancer types that were difficult to be predicted, it may be necessary to further select key genes for prediction so as to eliminate the interference of useless gene signatures. The mean AUCs and standard errors of AUCs on predicting the tumor stages and 3-year overall survivals were listed in the Additional file 1. The comparison results with RF and LR were shown in Additional file 3. The performance of RF was similar to that of SWT-CNN on predicting the tumor stages, and slightly better than that of SWT-CNN on predicting 3-year overall survival. In the subsequent analysis, we continued to investigate the utility of the representative features on selecting important genes and improving the prediction of such cancers. In addition, Kaplan-Meier survival analysis was conducted for all the cancer types. Figure 4 showed the survival curves of the patients in all the cancer types that were predicted to be high-risk or low-risk. The log-rank *p* values for all the cancer types were less than 0.0001, indicating that the survival times of the predicted high-risk patients were significantly different than those of the predicted low-risk patients.

### Identification of prognostic genes for further risk stratification

In the previous prediction, SWT-CNN model used all the genes as features for the clinical prediction. For each cancer type, we tried to apply our proposed scoring method to evaluating the importance of genes in the gene list and use the genes highly associated with the cancer to predict the overall survivals in the patients. We randomly selected 70% samples from the data set as the training set to build the model and left the rest samples as validation set. For each cancer type, the SWT-CNN model was firstly constructed and then, the representative features were extracted from the hidden layer of CNN to scoring the genes. Finally, the genes were ranked by their scores and the top *n* genes were used in the Cox proportional-hazards regression for the prediction of overall survival after treatments. We applied Kendal-Tau measure to compare top 100 genes in gene lists generated from the 5 bootstrap for 10 TCGA datasets (see Additional file 6). For example, the Kendal-Tau values are all more than 0.78 with corresponding *p* values less than 0.001 for the 5 gene lists in LGG, indicating the high stability of gene lists generated by the proposed SWT-CNN.

We took the gene expression data of OV as an example, for which the worst result was achieved by SWT-CNN in Fig. 3c. From the 274 samples, 191 samples were randomly selected as the training set and the original gene expression matrix *Y* (26, 270 genes × 191 samples) was constructed. The SWT-CNN had been run for 100 times on the training set and the best model was kept for extracting the representative features, which was a three dimensional matrix (191 samples × 3284 convolutional features × 64 channels) obtained by the treatment of pooling layer in CNN. Then, we averaged the data on 64 channels and obtained the representative feature matrix *X*, which contained 3284 features in rows and 191 samples in columns. According to our proposed method, the mapping coefficients from *X* to *Y* (matrix *B* with 26, 270 genes in rows and 3284 representative features in columns) were calculated. Finally, the matrix *B* was averaged by rows and a score vector with order 26, 270 × 1 was obtained, denoting the importance of the 26, 270 genes in the classification. The genes were ranked by their scores and the top 700 genes were used in the modeling procedures of univariate Cox regression and multivariate Cox regression. The genes significantly associated with the overall survival were listed in Table 3. In total, 67 genes were considered to be significantly associated with the 3-year overall survival of OV by univariate Cox regression and 11 genes were considered to be significant by the multivariate Cox regression. The genes selected by univariate and multivariate Cox regression for the other types of cancers were listed in Additional file 2. These genes might be considered as the diagnostic genes in the future studies.

**Table 3:** The genes considered to be significantly associated with the 3-year overall survivals of OV by the univariate Cox regression

Characteristics	***P***.Value
SACS	0.0002
SSC5D	0.0002
TSHR^a^	0.0003
CTD-2006C1.13	0.0004
LATS1	0.0008
HSPG2^a^	0.0008
AGPAT9	0.0019
STK38L	0.0032
CACNA1C	0.0033
AC005330.2^a^	0.0034
RP11-254F7.2	0.0047
MYH2	0.0048
ALDOA	0.0049
HIGD2A	0.0075
COL1A1	0.0101
ANAPC7^a^	0.0103
GIP	0.0110
BRD1	0.0117
MCL1	0.0126
IGDCC4	0.0137
FABP4^a^	0.0142
CHCHD10	0.0147
C12orf5	0.0148
COL3A1	0.0152
FAM196B	0.0171
CTD-2583A14.10	0.0181
DLX4	0.0182
ANKRD46	0.0183
ABHD15	0.0189
COX4I1^a^	0.0191
EPHB4	0.0202
RP5-1024G6.5	0.0204
RPL10	0.0218
GP9	0.0221
RPL15	0.0231
SLC34A2^a^	0.0243
LINC00891	0.0246
CD81	0.0247
B4GALT4	0.0253
BEST3	0.0254
ARHGAP5	0.0262
CCDC38	0.0263
RP11-77 K12.10^a^	0.0269
MMP2	0.0284
GLMN	0.0337
MAFA	0.0343
NCBP2^a^	0.0348
DOK6	0.0379
P2RY6^a^	0.0380
RP11-282O18.3	0.0381
FOLR1	0.0394
ORAI2	0.0411
FNBP1L	0.0412
NLGN2	0.0413
LL22NC03-2H8.4	0.0421
IER3IP1	0.0424
TRPC4	0.0427
RPS6	0.0429
RP11-894P9.2	0.0435
RPS25	0.0438
FTH1	0.0448
RP11-867G23.10^a^	0.0453
NPM2	0.0467
AP001372.2	0.0468
HOXD3	0.0469
XX-C283C717.1	0.0477
RGMB-AS1	0.0500

^a^marked the genes selected by multivariate Cox regression

**Fig. 5:**
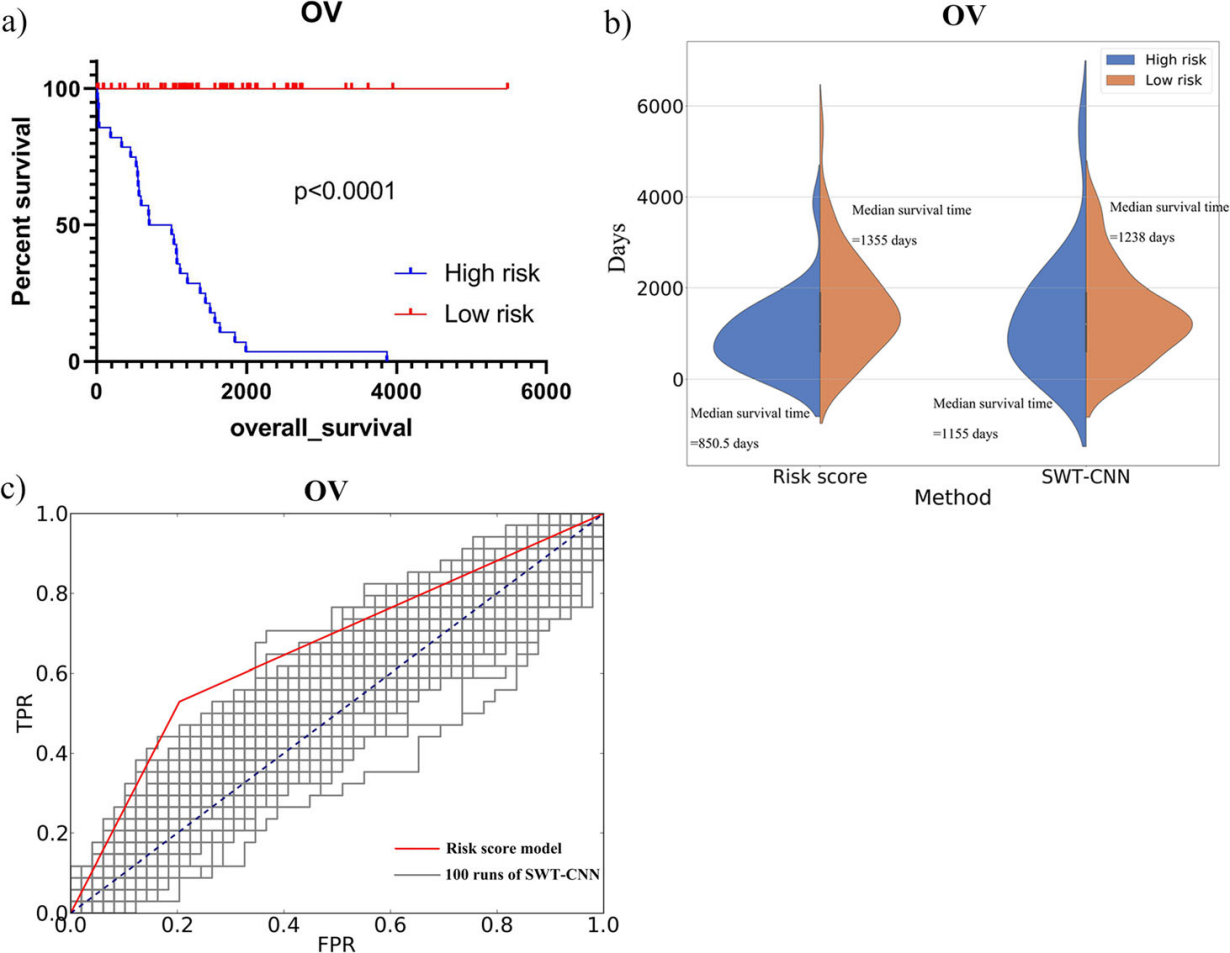
The results of risk score model for predicting the 3-year overall survivals of OV. **a** The survival curves of high-risk and low-risk patients in OV data set stratified by risk score model. **b** The distribution of survival times of high-risk and low-risk patients stratified by the risk score model and SWT-CNN. (c) The ROC curves achieved by the risk score model and 100 runs of SWT-CNN

**Fig. 6:**
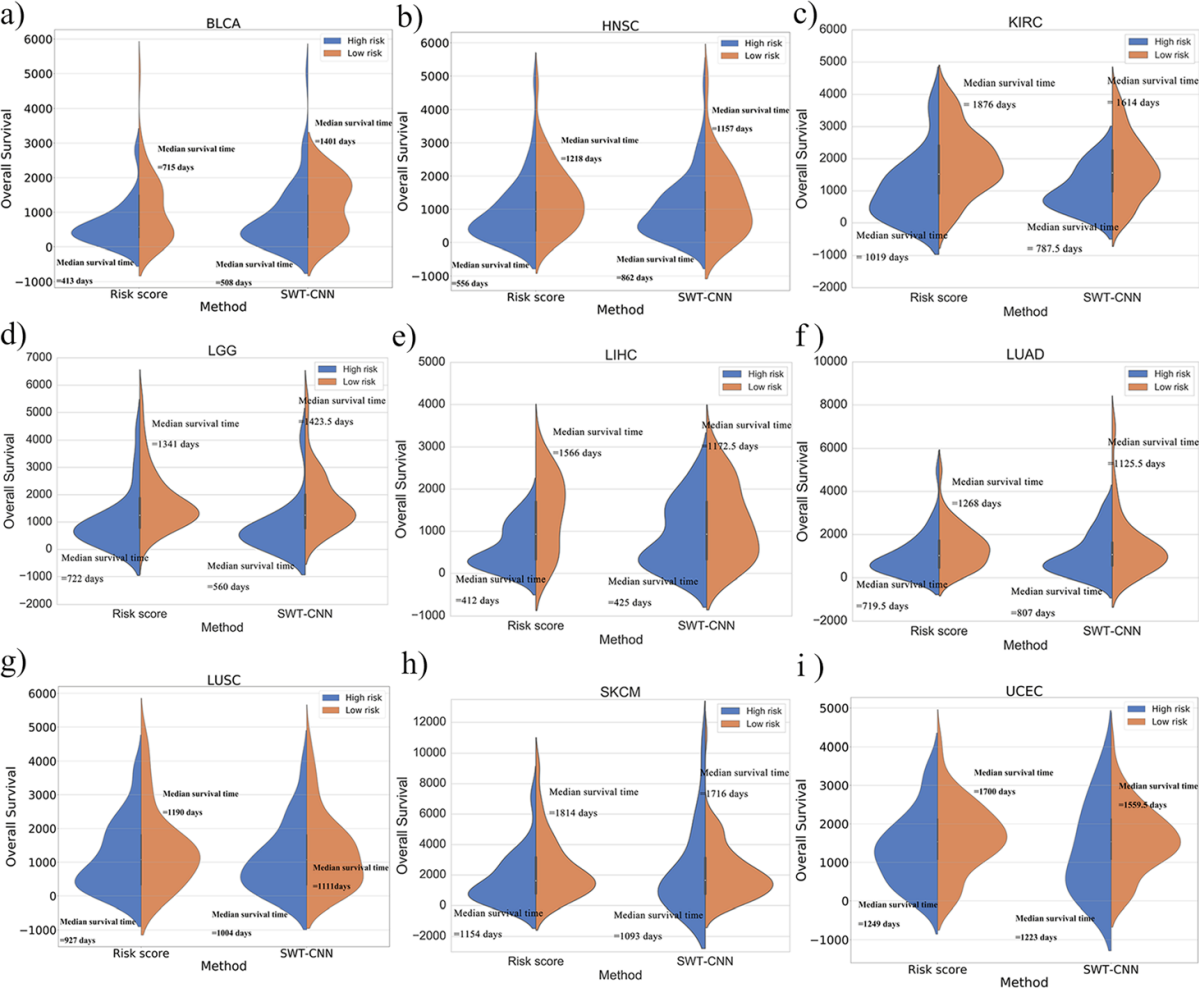
The distribution of survival times of high-risk and low-risk patients for the other cancer types stratified by the risk score model and SWT-CNN

**Fig. 7:**
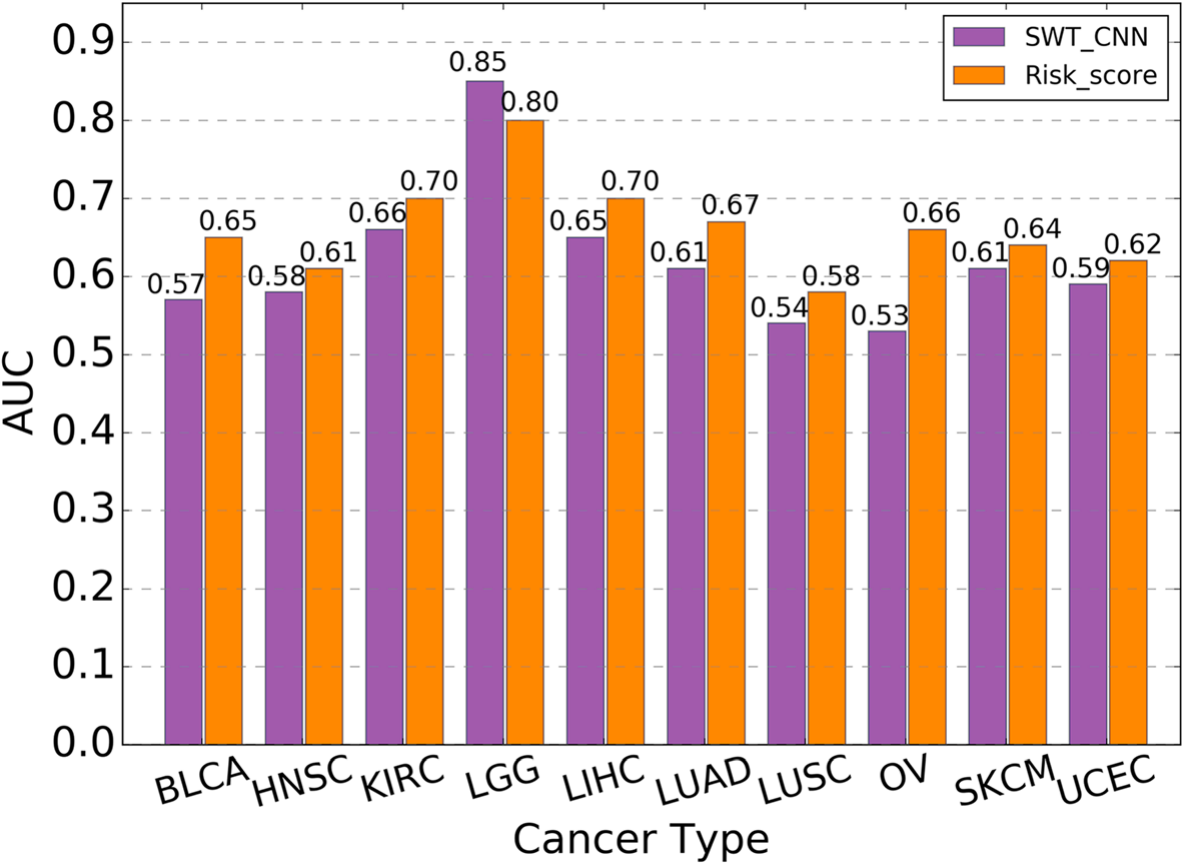
The AUCs achieved by risk score model and the mean AUCs achieved by 100 runs of SWT-CNN for predicting the 3-year overall survivals of all the data set

After assigning the risk score for each of the patients by multivariate Cox regression, the receiver operating characteristics curve (ROC) was employed on the training set to determine the cutoff of risk score for the stratification of the patients. The patients with the risk score higher than the cutoff were assigned to the high-risk group and the rest were assigned to the low-risk group [42]. The stratification model was validated by using the validation set. Figure 5 showed the stratification results for OV data set. It can be seen from the K-M survival curves (Fig. 5a) that there was a more significant difference (log-rank test *p* value < 0.0001) in overall survival time between the high-risk and low-risk patient groups divided by the risk scores. The median overall survival time for the high-risk and low-risk groups was 850.5 and 1355 days, respectively. Figure 5b showed the distribution of the survival time of the high- and low-risk groups divided by risk score and SWT-CNN. Compared with the results by SWT-CNN, the mean survival time of the high-risk patients predicted by risk score was lower than that predicted by SWT-CNN. The distribution of the overall survival time for other types of cancers was shown in Fig. 6. In general, the risk stratification for patients by risk score with the diagnostic genes was more accurate than that by SWT-CNN with all the genes. The median survival time of the high- and low-risk groups divided by risk score and SWT-CNN for all the cancer types were listed in Table 4. Using the same labels defined in the previous prediction step, we evaluated the prediction performance of the risk score model on predicting the 3-year overall survivals of all the data set. For OV data set, Fig. 5c showed the ROC curves achieved by the risk score model (AUC = 0.66) and 100 runs of SWT-CNN (mean AUC = 0.53). It can be seen that the prediction of 3-year overall survival was obviously improved by the risk score model with the diagnostic genes as features. Figure 7 showed the AUCs achieved by risk score model as well as the mean AUCs of SWT-CNN. In general, compared with the prediction results of SWT-CNN, the prediction performance of the risk score model for all the cancer types has been improved except for LGG, demonstrating that the representative features generated by CNN can be helpful for identifying the disease-related genes. More importantly, risk score model generated a relatively small gene set, which can provide a more precise set of candidate genes for the subsequent biological interpretation and experimental verification in clinics. For the data sets of OV, LUAD, LIHC and BLCA, the AUC increased by 0.13, 0.06, 0.05 and 0.08. To some extent, the prediction performance of the risk score model for the other cancer types had also been improved.

**Table 4:** Median survival time of the high-risk and the low-risk patients that divided by the risk score model and SWT-CNN

Type	Risk stratification	Median survival time	
		Risk Score	SWT-CNN
BLCA	Low Risk	715	1401
	High Risk	413	508
HNSC	Low Risk	1218	1157
	High Risk	556	862
KIRC	Low Risk	1876	1614
	High Risk	1019	787.5
LGG	Low Risk	1341	1423.5
	High Risk	722	560
LIHC	Low Risk	1566	1172.5
	High Risk	412	425
LUAD	Low Risk	1268	1125.5
	High Risk	719.5	807
LUSC	Low Risk	1190	1111
	High Risk	927	1004
OV	Low Risk	1355	1238
	High Risk	850.5	1155
SKCM	Low Risk	1814	1716
	High Risk	1154	1093
UCEC	Low Risk	1700	1559.5
	High Risk	1249	1223

## Discussion

Considering the fact that deep learning has been widely used in pattern recognition and started to be applied in cancer prognosis prediction, we proposed a method called SWT-CNN and thoroughly investigated the performance of the model on the clinical cancer prediction. In our study, the gene expression profiles of the patients were firstly decomposed into the wavelet coefficients by the stationary wavelet transform for the purpose of enhancing the weights of genes with relatively low expression levels in the gene list, and then were subsequently applied to the model construction and clinical prediction by using the convolutional neural network. In the modeling procedures, CNN algorithm can efficiently abstract the representative features from the gene expression patterns that highly associated with the cancer type by using a representation learning strategy, which has been considered to be superior to the conventional feature selection procedures. For the evaluation of the models, we followed the analytical pipeline in MAQC-II study [8] and used AUC as the metric to evaluate the model performance. Note that, for the prediction of continuous values related to the survival, Harrell’s c-index would be more suitable than AUC on assessing the performance of the models [43].

In general, the significant discrepancy in the prediction results mainly existed among different cancer types, which depended on the predictability of the clinical endpoints (Fig. 3a and c). In addition, the partition of data sets also has a certain impact on the prediction results, indicating that more samples should be added to improve the stability of the model (Fig. 3b and d). SWT-CNN showed superior prediction performance when comparing with SVM. For the cancer types that were easy to predict, both SWT-CNN and SVM achieved satisfied prediction results. The mean AUCs for predicting the tumor stage of KIRP and the 3-year overall survival of LGG achieved by SWT-CNN and SVM were higher than 0.80 (mean AUCs = 0.82 and 0.85, resp.) and 0.70 (mean AUCs = 0.71 and 0.74, resp.), respectively. However, for some cancer types, the prediction performance of SVM is obviously insufficient. When predicting the tumor stage and the 3-year overall survival of KIRC, the mean AUCs achieved by SVM were only 0.55 and 0.52, respectively, while the mean AUCs achieved by SWT-CNN were 0.74 and 0.66, respectively. For the cancer types that were difficult to predict, neither method can achieve satisfied results, even if the prediction results of SWT-CNN were slightly better than those of SVM. It is worth noting that almost all the gene expression-based prediction models are data dependent. To elucidate this point, we conducted the prediction of tumor stages and 3-year overall survivals of all the cancer types by using other two popular machine learning algorithms, namely random forest and logistic regression. The AUCs of predicting the tumor stages and 3-year overall survivals of all the cancer types were shown in the Additional file 3. It can be seen that the performance of random forest was comparable with that of the SWT-CNN and was superior to the performance of SVM and logistic regression. Therefore, except for the prediction accuracy, it is necessary to pay more attention to whether the model can generate an interpretable gene set for the subsequent downstream analysis.

For the gene expression-based prediction in clinics, people not only expect that the model performance is as good as possible, but also expect to obtain the interpretable gene features, which is not only convenient for subsequent biological analysis of cancer mechanisms, but also provides candidates for the discovery of valuable clinical diagnosis genes. To improve the prediction performance, more reliable candidate genes should be selected for model construction. Therefore, in this study, we also proposed a strategy to map the representative features in CNN to the original genes and weighted them with the scores. The higher score of a gene indicated that its expression level in the genome was more important for the clinical prediction. Considering the fact that CNN algorithm tends to give heavy weights to those features with large values while ignoring some small ones, we introduced SWT to enhance the significance of genes with relatively low expression levels in the gene list and make CNN algorithm weight the genes objectively. The prediction results of 3-year overall survival by using CNN with and without SWT were shown in Additional file 4. The performance of the models was comparable. However, when extracting the genes from the representative features generated by CNN alone and ranking them by their scores, the mitochondrial function related genes, namely MT-CO1, MT-ND4, MT-CO3, MT-ND1, MT-ND3, MT-CO2, MT-ND2, MT-ATP6, MT-ND4L and MT-CYB, were ranked in the top of the gene list in most of the models because their expression levels were pretty higher than those of other genes. When transforming the gene expression profile by using SWT before CNN modeling, the candidate genes can be correctly ranked in the gene list. By using these genes as features in the univariate and multivariate Cox regression, we finally established the risk score models for predicting the 3-year overall survivals of all the data set. For the data sets of BLCA, LIHC, LUAD and OV, the performance of risk score models (AUCs = 0.65, 0.70, 0.67 and 0.66, resp.) was higher than that of SWT-CNN (mean AUCs = 0.57, 0.65, 0.61 and 0.53, resp.). Note that, for the other cancer types, the prediction performance of risk score model was only slightly improved when comparing with that of SWT-CNN. The reason may be that the representative features cannot extract the gene expression patterns effectively. In addition, by using the candidate genes generated by the risk score model as features, we reconstructed the SVM models for predicting the 3-year overall survivals of all cancer types. The results showed that the performance of SVM is improved to some extent after using the new features when comparing with the original SVM models (Additional file 5), indicating the effectiveness of candidate genes generated from the representative features of CNN. In order to facilitate interpretation, we used a single-layer network for all data sets. In fact, for some cancer types that were hard to predict, we can appropriately increase the number of layers to ensure the effectiveness of representative feature extraction. More detailed researches on the adjustment of CNN parameters for specific endpoints can be conducted in the future work.

To further validate the function of the genes that were selected as features in univariate and multivariate Cox regression modeling, we mapped the genes to the Cancer Gene Census (CGC) data set in the Catalogue Of Somatic Mutations In Cancer (COSMIC release v90) database [44]. For the data set of OV, 67 genes were identified by univariate Cox regression to be significantly associated with the cancer (Table 3) and seven of them, namely *COL1A1*, *COL3A1*, *RPL10*, *ARHGAP5*, *LATS1*, *TSHR* and *SLC34A2*, were found in the CGC data set. Genes *COL1A1* and *COL3A1* are demonstrated that played an unfavorable role in the development of ovarian cancer, and could be considered as the prognostic genes of OV [45]. Moreover, *COL1A1* has also been found to be positively related with the degree of invasion, metastasis, and advanced stages of gastric cancer [45, 46]. *COL3A1* can also be considered to be a potential biomarker for colon cancer [47], breast cancer [48] and brain tumor [49]. *ARHGAP5* was identified as an oncogene which can promote tumor metastasis and proliferation [50, 51]. It was also proved that *ARHGAP5* could be a prognostic marker of gastric cancer [50] and the expression level of it was associated with invasive and migrative ability of nasopharyngeal carcinoma cells [51]. *LATS1* is one of family proteins of large tumor suppressor (LATS) and has been proposed to be a tumor suppressor [52]. It had been found that the expression level of *LATS1* had decreased in serous ovarian cancer patients but this gene highly expressed in normal ovarian tissue [52, 53]. *SLC34A2* was proved to have an evident effect in the progression of several types of cancers, such as in ovarian cancer [54, 55], breast cancer and non-small cell lung cancer [56, 57]. The gene fusion of *SLC34A2* and *ROS1* played an important role in the progression of non-small cell lung cancer [58]. In addition, genes *LRP1B* and *CCR4* were identified by univariate Cox regression from the BLCA and HNSC data sets (Additional file 2), respectively, which were reported in the COSMIC database. *LRP1B* is one of the top ten genes mutated in human cancers [59, 60] and might be a potential contributor to the emergence of chemotherapy resistance [59]. Gene *CCR4* was reported to be associated with adult T-cell leukaemia and lymphoma in the COSMIC database. The ligands for *CCR4* are produced by tumor cells or the microenvironment, and can attract CCR4-expressing T regulatory cells and create a good survive environment for tumor cells [61]. Moreover, genes *SMARCD1* were identified by univariate Cox regression from both the KIRC and LIHC data sets, and gene *TSHR* were identified by univariate Cox regression from the LUAD data sets (Additional file 2). These results indicated that that our proposed scoring method can effectively screen the diagnostic genes for clinical cancer prediction by using representative features to evaluate the importance of genes.

In addition, some caveats were still necessarily discussed: 1) The performance of SWT-CNN was comparable to CNN (Fig. 1 in Additional file 4), indicating that the main contribution of SWT in the model was to enhance the significance of genes with relatively low expression levels. 2) Only a small proportion of genes identified by our strategy were included in the current version of the COSMIC database, and further experimental verification of the causal relationship of remaining genes and cancer etiology is needed. 3) To facilitate the evaluation of the performance of SWT-CNN, SVM, RF, and LR, we categorized the patients into two groups (high/low-risk groups) according to their tumor stages or survival times. The prediction results of multivariate Cox regression were also dichotomized by using a risk score cutoff. In fact, for the prediction of the survival, both deep learning-based algorithms *(*e.g.*,* DeepSurv [43]) and Cox regression can directly use continuous values (e.g., survival time) for modeling. The prediction results can be evaluated by AUC [62] or c-index [63]. 4) Compared to AUC, c-index is a more statistically stringent performance metric for evaluating the survival models. Specifically, the c-index measure inspects the consistency of predicted and the actual labels (i.e., clinical outcomes), but also inspects the correlation between the predicted values and their survival time. For the binary classification, the result of the c-index is almost equivalent to that of AUC, e.g., evaluating the performance of logistic regression in binary classification. 5) As a classical signal processing method, Fourier transform can also obtain the Fourier coefficients by convoluting the original signal with Fourier functions. Compared with Fourier transform, wavelet transform has some advantages [64–66]. In this study, we used wavelet transform to decompose the gene expression profile, because the low frequency part of the wavelet coefficients was the approximation of the original signal, which can facilitate us to map the score matrix back to original gene list. The Fourier coefficients can only reflect the frequencies of sin/cos functions and it is difficult to match the Fourier coefficients with the original genes. Further researches can also explore whether it is possible to combine Fourier transform with CNN to identify the disease-related genes.

## Conclusions

In our study, we proposed a gene expression-based method called SWT-CNN as an alternative for stratifying the prognostic risk for cancer patients and thoroughly investigated the performance of the model in the large data sets. Our results indicated that SWT-CNN can be an excellent tool for risk stratification in cancers. When evaluating the genes by using the representative features in CNN, the diagnostic genes that were highly associated with the cancers can be effectively identified and used as features for improving the prediction performance of the models. In addition, these diagnostic genes can also be helpful for better understanding the mechanism of the diseases.

## Methods

### Data sets

The RNA-sequencing data in FPKM format as well as the clinical information of the patients were downloaded from The Cancer Genome Atlas (TCGA) database [32] (https://portal.gdc.cancer.gov/). The summarized fragments per kilobase million (FPKM) of 60,483 transcripts (data in level 3) were firstly mapped to the unique genes by using the comprehensive gene annotation file (ftp://ftp.ebi.ac.uk/pub/databases/gencode/Gencode_human/release_22/gencode.v22.annotation.gtf.gz). In total, the expression data of 34,534 unique protein coding genes and lncRNA genes were generated for the subsequent predictive model construction. We removed the genes, for which the expression levels were zero in over half of the patients [67]. As a result, a subset of genes was kept for the subsequent analysis. We downloaded all the data sets from TCGA. After data processing, the gene expression data of twelve cancer types, namely BLCA, BRCA, COAD, HNSC, KIRC, KIRP, LIHC, LUAD, LUSC, SKCM, STAD, THCA were used for the prediction of tumor stage, and the data of ten cancer types, namely BLCA, HNSC, KIRC, LGG, LIHC, LUAD, LUSC, OV, SKCM, UCEC were used for the prediction of 3-year overall survival.

To investigate the model performance on predicting clinical endpoints, we categorized the patients into two compared groups with different clinical information. For tumor stage prediction, the patients with the tumor stages of III and IV were categorized into the high-risk group and those with the stages I and II were categorized into the low-risk group. As for 3-year overall survival prediction, the patients, whose survival times were less than 3 years, were categorized into the high-risk group, and the rest were categorized into the low-risk group. Note that the patients, whose survival times were less than 3 years but still alive, were removed from the data sets. For both tumor stage and 3-year overall survival, the patients in high-risk group were considered as the positive samples. The number of samples and the proportion of positive and negative samples in each cancer data set were listed in Tables 1 and 2.

### Support vector machine

Support vector machine (SVM) [68, 69] is a popular machine learning algorithm, which was firstly proposed by Vapnik [70] and has been widely used in binary classification for decades. SVM can well classify the samples via projecting the samples to the higher-dimensional space from the original space and searching for an optimal hyperplane for classifying the samples. Before model construction, the genes were filtered by using Student’s *t*-test and a fold change cutoff. Only the genes, for which the *p* values < 0.05 and the absolute values of fold change > 2, were kept for the subsequent modeling. In our study, we used *rbf* as the kernel function in SVM and optimized the parameters (*c and gamma in kernel function*) by a grid search approach. In order to reduce the impact of data partitioning on results, we randomly selected 70% samples as the training set and validated them by using the rest 30% samples. This procedure had been run for 100 times. The SVM modeling procedure was conducted in python 2.7 by using the *sklearn* package.

### Stationary wavelet transform

The concept of wavelet was firstly introduced by Morlet and Grossmann [71] and had been successfully applied in signal processing field for decades. In a square integrable space *L*^*2*^*(ℝ)*, the wavelet function is defined as:

1 $$ {\upvarphi}_{\mathrm{a},\mathrm{b}}\left(\mathrm{t}\right)=\frac{1}{\sqrt{\mathrm{a}}}\upvarphi \left(\frac{\mathrm{t}-\mathrm{b}}{\mathrm{a}}\right),\mathrm{a},\mathrm{b}\in \mathrm{R} $$

Where *a* and *b* represent the scale and translation parameters, respectively. A wavelet family can be generated by means of translations and dilations of φ. The continuous wavelet transform procedure can be described by a following equation:

2 $$ {\mathrm{F}}_{\mathrm{CWT}}\left(\mathrm{a},\mathrm{b}\right)=\frac{1}{\sqrt{\mid \mathrm{a}\mid }}{\int}_{-\infty}^{+\infty}\mathrm{f}\left(\mathrm{t}\right)\upvarphi \left(\frac{\mathrm{t}-\mathrm{b}}{\mathrm{a}}\right)\mathrm{dt} $$

Where *f(t)* is the original signal. It can be seen that the transformed signal (wavelet coefficients) *F*_*CWT*_*(a,b)* is the result of convolution between the original signal and the wavelet function. It is also a function of scale parameter *a* and translation parameter *b*. The inverse continuous wavelet transform can be easily conducted by calculating the convolution of transformed signal and the wavelet function:

3 $$ \mathrm{f}\left(\mathrm{t}\right)=\frac{1}{{\mathrm{C}}_{\upvarphi}^2}{\int}_{-\infty}^{+\infty }{\int}_{-\infty}^{+\infty }{\mathrm{F}}_{\mathrm{C}\mathrm{WT}}\left(\mathrm{a},\mathrm{b}\right)\frac{1}{{\mathrm{a}}^2}\upvarphi \left(\frac{\mathrm{t}-\mathrm{b}}{\mathrm{a}}\right)\mathrm{dbda} $$

where *C*_*φ*_ is the admissibility constant, which depends on the chosen wavelet function.

In general, the eq. (1) is discrete by using:

4 $$ \mathrm{a}={2}^{-\mathrm{j}},\mathrm{b}={2}^{-\mathrm{j}}\mathrm{k}\ \left(\mathrm{j},\mathrm{k}\in \mathrm{Z}\right) $$

Then, the Discrete Wavelet Transform (DWT) can be defined as:

5 $$ {\mathrm{F}}_{\left(\mathrm{DWT}\right)}\left(\mathrm{j},\mathrm{k}\right)={2}^{\mathrm{j}/2}{\int}_{-\infty}^{+\infty}\mathrm{f}\left(\mathrm{t}\right)\upvarphi \left({2}^{\mathrm{j}}-\mathrm{k}\right)\mathrm{dt} $$

After transformation, the original signal has been decomposed into the wavelet coefficients of the first layer, which represents the information of the low frequency part (approximate profiles) and the high frequency part (details) in the original signal, respectively. Then, the low frequency part can be further decomposed into the wavelet coefficients of the second layer and so on. As the number of decomposition layers increases, the degree of signal approximation increases. The loss of information is also increasing. We tested the prediction results by using the wavelet coefficients decomposed from 3 to 5 layers respectively (data not shown), and found that it had little influence on the prediction performance of the models. So, we chose a smaller number of decomposition layers to keep the original information as much as possible. In most cases, e.g. in the chemical signal processing, the low frequency part of the wavelet coefficients is the approximation of original signal and can reflect the profile of original signal to a certain extent. The high frequency part of the wavelet coefficients is usually considered to be related to the noise of the original signal. This is the reason why the high-frequency part of the coefficients is usually discarded when using the wavelet transform for signal denoising. In our study, we only used the low frequency part of the wavelet coefficients for the subsequent analysis. In addition, in order to maintain the number of features, we used stationary wavelet transform (SWT), also known as undecimated wavelet transform, which does not decimate coefficients at every transformation level. It is a translation-invariance modification of the DWT [72]. Due to the up-sampling operation of the filter coefficients, the SWT has the advantage of being shift-invariant compared with DWT [73, 74].

In this study, SWT can make generalization of the expression profiles of grouped genes and denoise the gene expression signal. When the gene expression profile was decomposed into the wavelet coefficients, we kept the low frequency part of the coefficients to obtain a cleaner signal, which was the approximation of original gene expression profile. Meanwhile, the difference in expression levels of different genes will be reduced in the process of approximation, which is conducive to generating the representative features by CNN. The gene expression profile of a patient was firstly decomposed into a certain layer by the stationary wavelet transform and then, the wavelet coefficients were subsequently input into the convolutional neural network. For instance, a gene expression matrix *X* contains *n* samples in rows and *p* genes in columns. The wavelet transform will decompose the gene expression data by samples. For each sample, the gene expression profile is a vector with order *1* × *p* (1 sample × *p* genes). If we decompose the profile into m layers, the wavelet coefficients matrix will be *m* × *p* (*m* layers × *p* wavelet coefficients). This decomposition procedure has been repeated for *n* times and the gene expression profiles of all samples have been transformed to the wavelet coefficients. As a result, the final wavelet coefficients matrix should be *n* × *p* × *m* (*n* samples × *p* wavelet coefficients × *m* layers). Subsequently, the wavelet coefficients matrix is input into CNN for modeling. Note that the wavelet coefficients are only the result of mathematical transform, which is the approximation of the original gene expression profile but cannot be directly associated with the biological meaning of the genes. Decomposing the signal with different wavelet functions may obtain different wavelet coefficients, it is necessary to investigate the impact of the wavelet coefficients calculated by different wavelet functions on the predictive models. After decades of development, many wavelet functions have been proposed for signal processing. Here, we chose four most commonly used wavelet families to test. Considering that there is little difference in wavelet basis functions in the same family, we selectively chose three wavelet basis functions from each family. Consequently, twelve commonly used wavelet functions were chosen and examined in this study including *Daubechies* wavelet family (*db1*, *db3* and *db5*), *Coiflets* wavelet family (*coif1*, *coif3* and *coif5*), biorthogonal wavelet family (*bior3.1*, *bior3.3* and *bior3.5*) and *symlets* wavelet family (*sym2*, *sym4* and *sym6*). The number of decomposition layers was set to 3. The wavelet decomposition procedures were conducted with a python package called PyWavelets [75]. To choose the optimal wavelet function, for each cancer type, we randomly selected 70% samples as training set and used the rest samples as the validation set. The twelve wavelet functions were separately used to decompose the gene expression profile of the sample and the wavelet coefficients were input into the CNN for modeling by using the training set. The validation set was used to evaluate the performance of the models. It can be decided which wavelet function combined with CNN was optimal for the current cancer type.

### Convolutional neural network

**Fig. 8:**
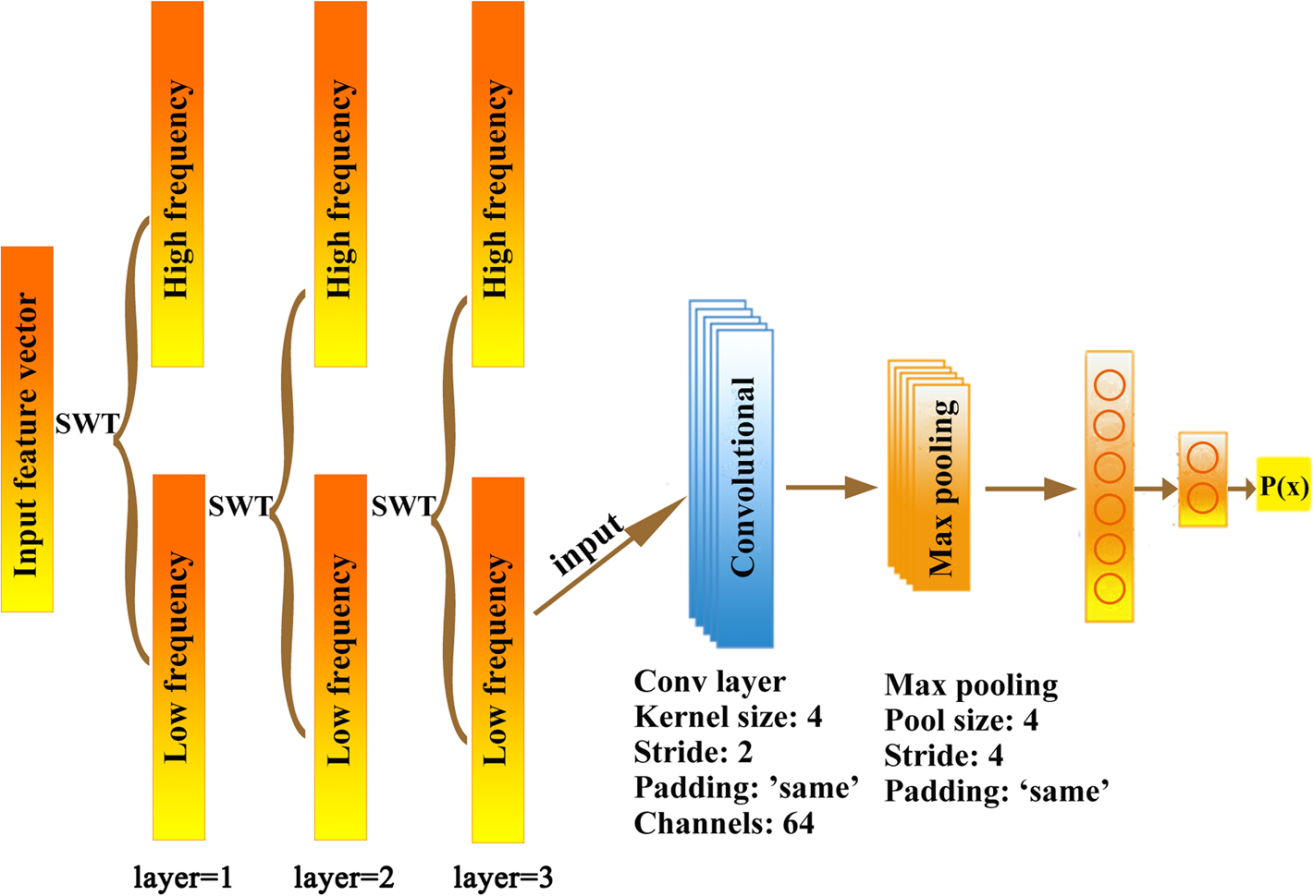
The architecture of the SWT-CNN model in our study

As one of the classical deep learning algorithms, convolutional neural network (CNN) [76, 77] is widely used in image processing. Similar to the conventional neural network, CNN includes an input layer, an output layer and a number of hidden layers. Among the hidden layers, CNN usually involves the convolutional layers and pooling layers, which can efficiently reduce the connections between the neurons and extract the features from the original image, respectively. In our study, we constructed the CNN models involving an input layer, a convolutional layer, a pooling layer, a full connective layer and an output layer. The architecture of the CNN model and the used parameters were shown in Fig. 8. The wavelet coefficients matrices of the patients were directly input into the CNN models for classification. The functions for optimizer, loss, activation and output were separately set to *RMSprop*, *binary_crossentropy*, *relu* and *softmax*. All the calculations of CNN modeling were conducted in python 2.7 by using the *tensorflow* and *keras* packages. Similarly, we randomly selected 70% samples as the training set to construct the models and validated them by using the rest 30% samples. The sampling procedure had been repeated for 100 times. In addition, The Kaplan-Meier survival analysis was applied in evaluating the stratification of the patients. The calculation was conducted in GraphPad Prism 8 software (https://www.graphpad.com/scientific-software/prism/).

### Scoring approach for evaluating gene importance

We proposed a scoring approach to identify the diagnostic genes for improving the performance of prediction. For each cancer type, 70% samples were randomly selected as training set, which was firstly used to construct the SWT-CNN model. The modeling procedure had been repeated for 100 times and the best model was kept for extracting the representative features. We extracted the coefficients after the treatment of pooling layer of the best model and averaged the coefficients for all channels to obtain the representative feature matrix *X*. Then, according to the principle of least squares, we calculated the scores for all genes as follows:

6 $$ \mathrm{B}=\mathrm{Y}\bullet {\mathrm{X}}^{\mathrm{T}}{\left(\mathrm{X}\bullet {\mathrm{X}}^{\mathrm{T}}\right)}^{-1} $$

where matrix *Y* was the wavelet coefficient matrix that input into the CNN model, and the result *B* was the score matrix for the wavelet coefficients with the number of coefficients in rows and the number of representative features in columns. Because the wavelet coefficients were the approximation of original gene expression profile, the score matrix of wavelet coefficients can also be regarded as the score matrix of genes. Then, we averaged each row of matrix *B* and obtained the scores for all genes, which indicated the importance of the genes to the cancer.

### Cox proportional-hazards regression

We extracted the top *n* genes ranked by the scores for the Cox proportional-hazards regression. The number *n* was tested from 100 to 1000 genes with a step of 100 and the optimal value was determined by using the training set. Univariate Cox regression was conducted by using the expression data of the top *n* genes combined with patient survival time for gene selection (*p* < 0.05). Then, the selected genes were used in the multivariable Cox regression. Note that, for the limited sample size and larger gene set, the multivariable Cox regression may be unstable and cannot converge. The penalized Cox regression [78] should be used instead. Finally, the genes significantly associated with the overall survival (*p* < 0.05) were considered as the diagnostic genes. To use these genes as features for the prediction of 3-year overall survival, we calculated the risk scores for the patients and summarized them as the probability of overall survival from the cancer data set as follow [42, 79, 80]:

7 $$ \mathrm{Risk}\ \mathrm{score}={\upbeta}_1{\mathrm{x}}_1+{\upbeta}_2{\mathrm{x}}_2+{\upbeta}_3{\mathrm{x}}_3+\dots +{\upbeta}_{\mathrm{N}}{\mathrm{x}}_{\mathrm{N}} $$

where *x*_*i*_ is the gene expression value of the *i*^th^ gene and β is the corresponding Cox coefficient. Then, receiver operating characteristics curve (ROC) was employed on the training set to determine the optimal cut-off points for classification [81]. And the cut-off was used to stratify patients into low- and high-risk groups in the validation set.

The source code can be downloaded from GitHub (https://github.com/zyrr183/TCGA_SWT-CNN-Risk-score-Method).

## Supplementary information

**Additional file 1.** The mean AUCs and standard errors of AUCs on predicting the tumor stages and 3-year overall survivals.

**Additional file 2.** Results of the univariate Cox regression. Tables contain the genes considered to be significantly associated with the 3-year overall survivals of all the data set by the univariate Cox regression.

**Additional file 3.** The performance of SWT-CNN, SVM, random forest and logistic regression on predicting the tumor stages and the 3-year overall survivals of all cancer types.

**Additional file 4.** The performance of CNN algorithm with and without SWT on predicting 3-year overall survival of all the cancer types.

**Additional file 5.** The performance of the models on predicting 3-year overall survivals of all cancer types.

**Additional file 6.** Kendal-Tau values of gene lists generated from the 5 bootstrap for 10 TCGA datasets.

## Data Availability

The sample data and the source code used in our study can be accessed from GitHub (https://github.com/zyrr183/TCGA_SWT-CNN-Risk-score-Method). The full data sets can be freely downloaded from The Cancer Genome Atlas (TCGA) database (https://portal.gdc.cancer.gov/). The datasets supporting the conclusions of this article are included within the article and its additional files.

## Abbreviations

AUC: The Area under the receiver operating characteristic curve
BLCA: Bladder urothelial carcinoma
BRCA: Breast invasive carcinoma
CNN: Convolutional neural network
COAD: Colon adenocarcinoma
HNSC: Head and neck squamous cell carcinoma
KIRC: Kidney renal clear cell carcinoma
KIRP: Kidney renal papillary cell carcinoma
LGG: Brain lower grade glioma
LIHC: Liver hepatocellular caa
LUAD: Lung adenocarcinoma
LUSC: Lung squamous cell carcinoma
OV: Ovarian serous cystadenocarcinoma
SKCM: Skin cutaneous melanoma
STAD: Stomach adenocarcinoma
SVM: Support vector machine
SWT: Stationary wavelet transform
TCGA: The Cancer Genome Atlas
THCA: Thyroid carcinoma
UCEC: Uterine corpus endometrial carcinoma
