# Supplementary material for: Uncovering the prognostic gene signatures for the improvement of risk stratification in cancers by using deep learning algorithm coupled with wavelet transform
Source: BMC Bioinformatics. 2020 May 19;21:195. doi: 10.1186/s12859-020-03544-z (PMC7236453; doi:10.1186/s12859-020-03544-z)
Supplement: Supplementary file 3 — Additional file 3. The performance of SWT-CNN, SVM, random forest and logistic regression on predicting the tumor stages and the 3-year overall survivals of all cancer types. [file 12859_2020_3544_MOESM3_ESM.pdf]

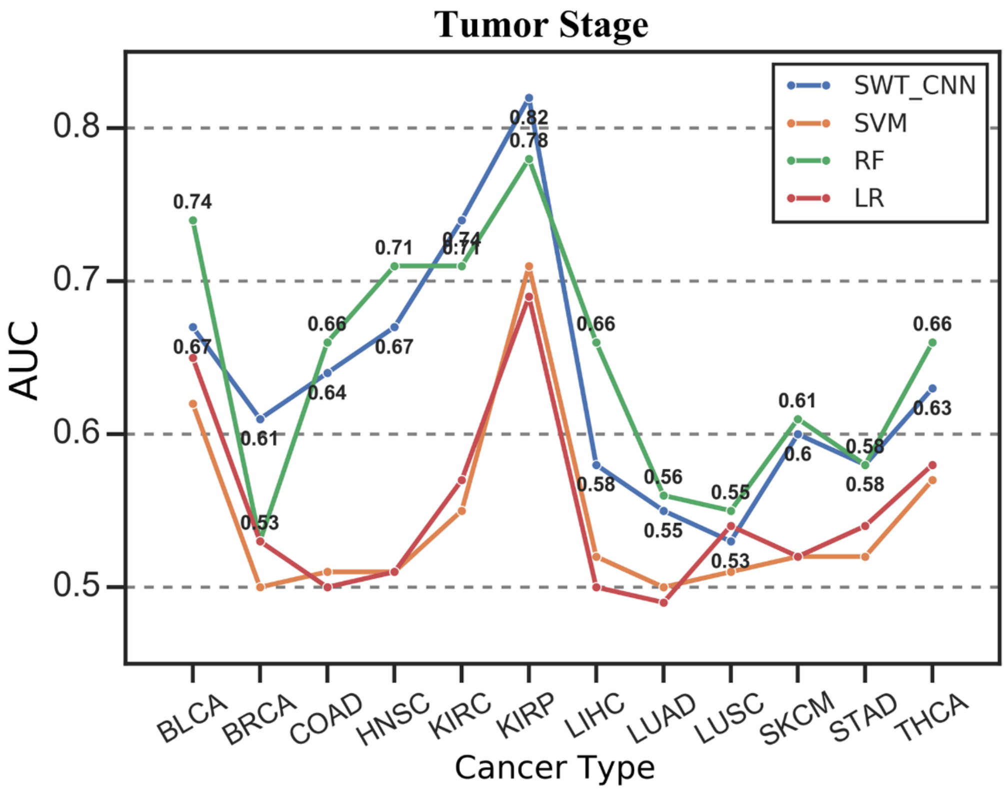

Figure 1. The performance of SWT-CNN, SVM, random forest and logistic regression on predicting the tumor stages of all cancer types.

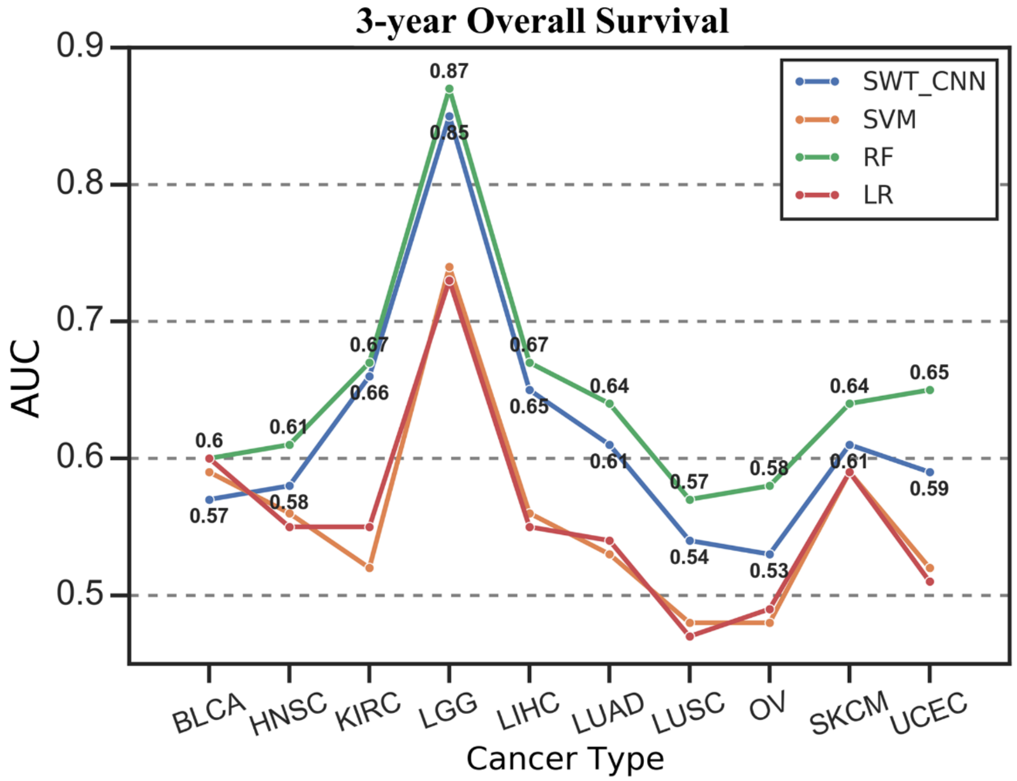

Figure 2. The performance of SWT-CNN, SVM, random forest and logistic regression on predicting the 3-year overall survivals of all cancer types.
