# Supplementary material for: Uncovering the prognostic gene signatures for the improvement of risk stratification in cancers by using deep learning algorithm coupled with wavelet transform
Source: BMC Bioinformatics. 2020 May 19;21:195. doi: 10.1186/s12859-020-03544-z (PMC7236453; doi:10.1186/s12859-020-03544-z)
Supplement: Supplementary file 4 — Additional file 4. The performance of CNN algorithm with and without SWT on predicting 3-year overall survival of all the cancer types. [file 12859_2020_3544_MOESM4_ESM.pdf]

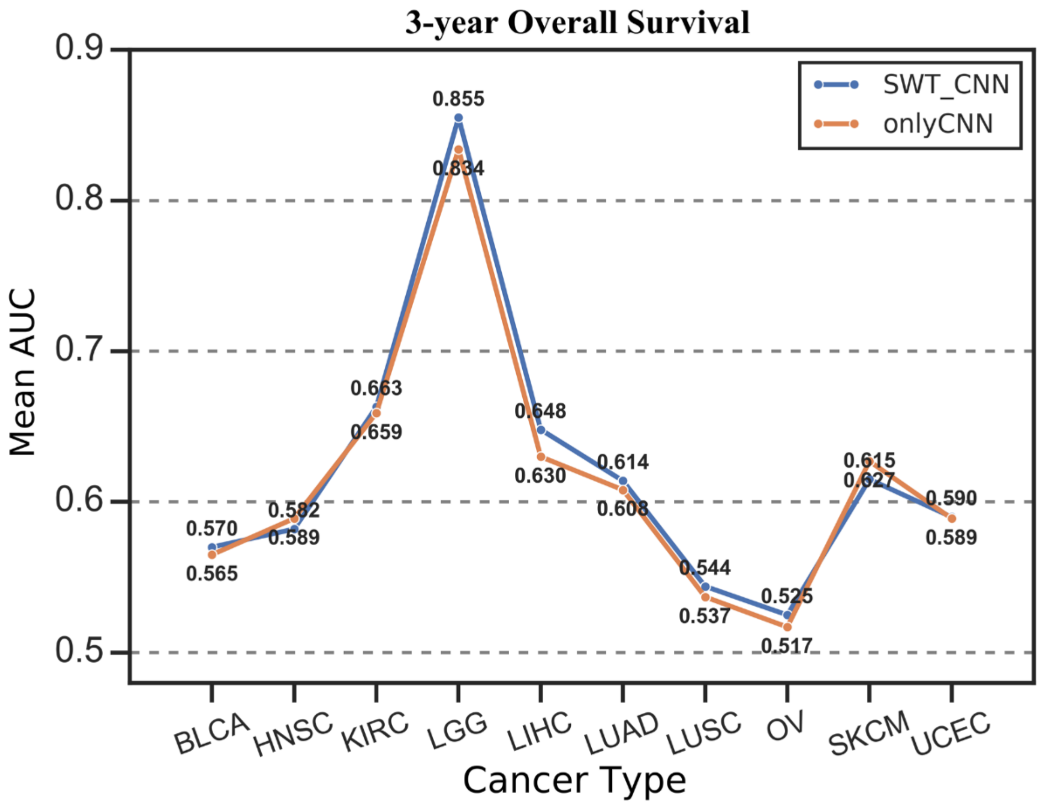

Figure 1. The performance of CNN algorithm with and without SWT on predicting 3-year overall survival of all the cancer types.
