# Supplementary figures and images for: Uncovering the prognostic gene signatures for the improvement of risk stratification in cancers by using deep learning algorithm coupled with wavelet transform
Source: BMC Bioinformatics. 2020 May 19;21:195. doi: 10.1186/s12859-020-03544-z (PMC7236453; doi:10.1186/s12859-020-03544-z)

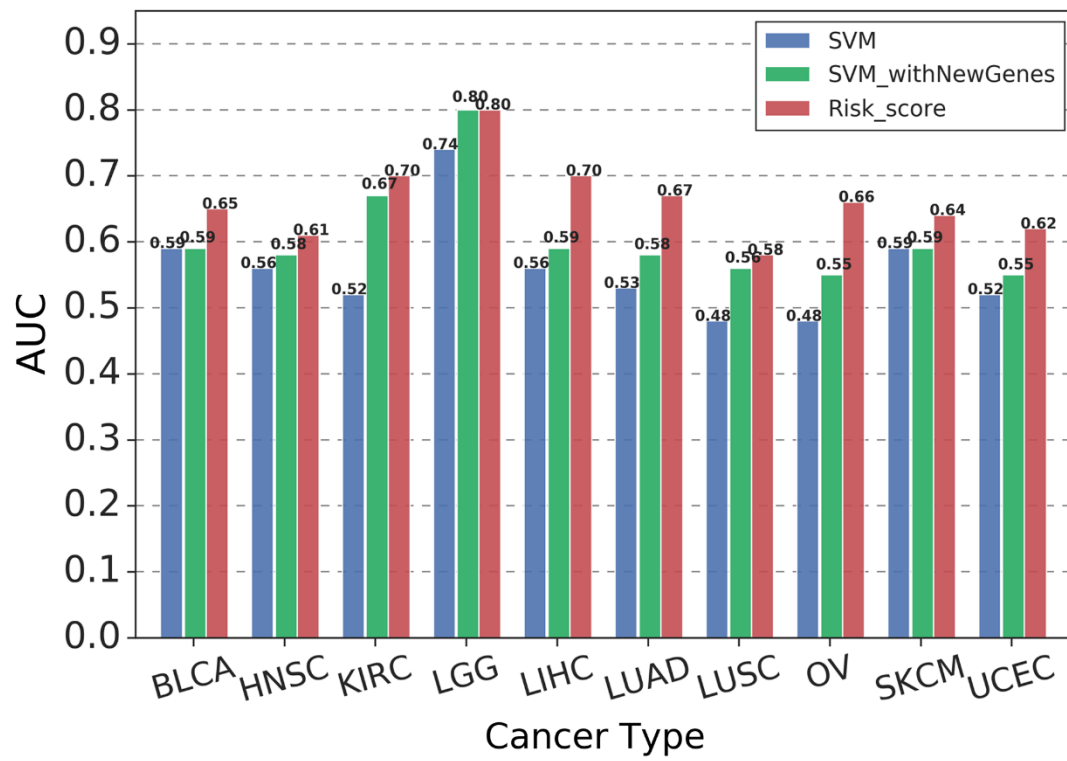

Figure 1. The performance of the models on predicting 3-year overall survivals of all cancer types

Supplement: Supplementary file 5 — Additional file 5. The performance of the models on predicting 3-year overall survivals of all cancer types. [file 12859_2020_3544_MOESM5_ESM.pdf]

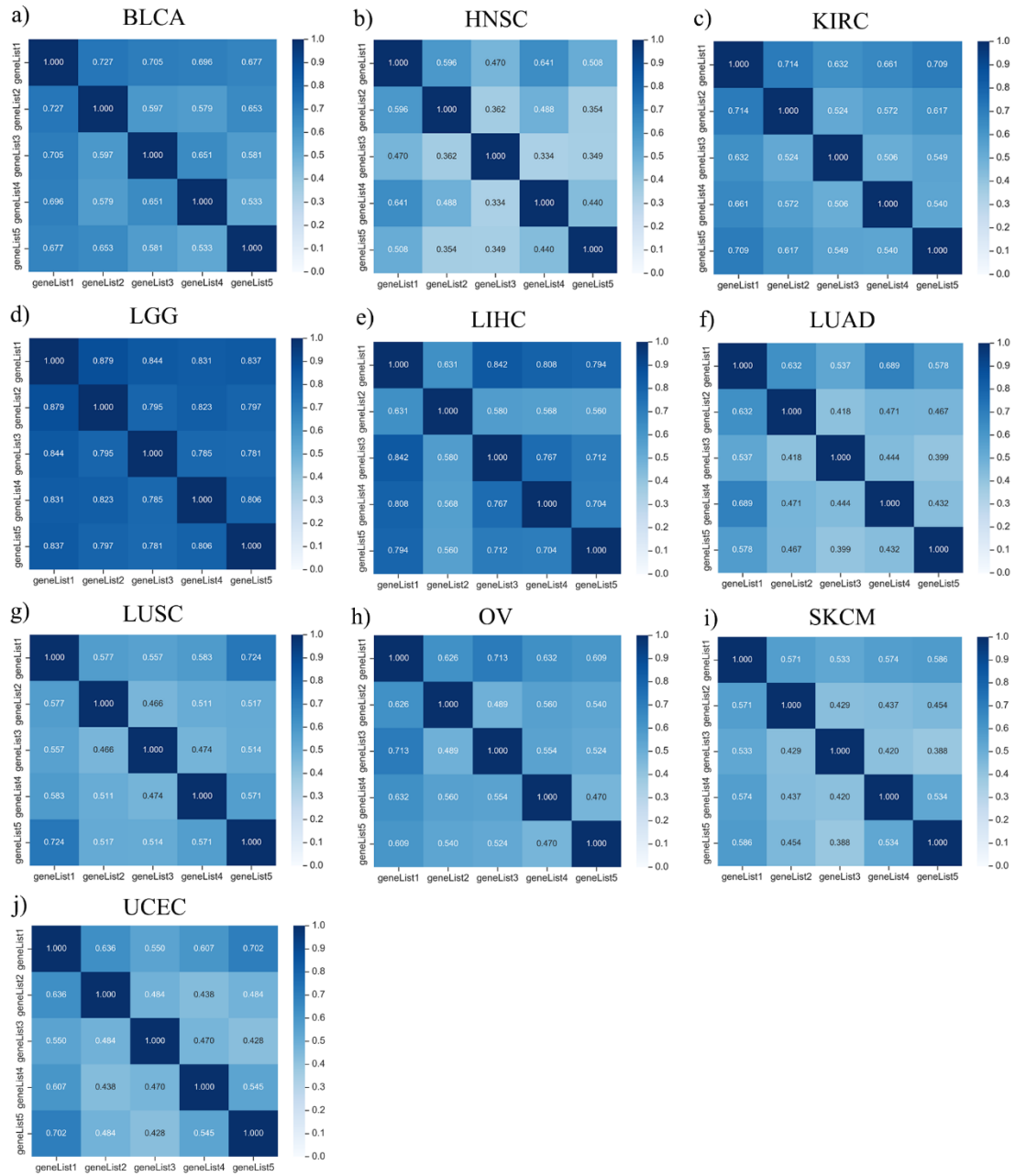

Figure 1. Kendall-Tau values of gene lists generated from the 5 bootstrap for 10 TCGA datasets

Supplement: Supplementary file 6 — Additional file 6. Kendal-Tau values of gene lists generated from the 5 bootstrap for 10 TCGA datasets. [file 12859_2020_3544_MOESM6_ESM.pdf]
